# Thermal Boundary Characteristics of Homo-/Heterogeneous Interfaces

**DOI:** 10.3390/nano9050663

**Published:** 2019-04-26

**Authors:** Koen Heijmans, Amar Deep Pathak, Pablo Solano-López, Domenico Giordano, Silvia Nedea, David Smeulders

**Affiliations:** 1 Energy Technology, Department of Mechanical Engineering, Eindhoven University of Technology, 5600 MB Eindhoven, The Netherlands; k.heijmans@tue.nl (K.H.); simplyamar06@gmail.com (A.D.P.); d.m.j.smeulders@tue.nl (D.S.); 2 Departmento de Fisica Aplicada, ETSIAE, Universidad Politécnica de Madrid, 28040 Madrid, Spain; Pablo.Solano@upm.es; 3 ESA—Estec, Keplerlaan 1, 2201 AZ Noordwijk, The Netherlands; dg.esa.retired@gmail.com

**Keywords:** ReaxFF, interface, thermal boundary resistance, Kapitza resistance

## Abstract

The interface of two solids in contact introduces a thermal boundary resistance (TBR), which is challenging to measure from experiments. Besides, if the interface is reactive, it can form an intermediate recrystallized or amorphous region, and extra influencing phenomena are introduced. Reactive force field Molecular Dynamics (ReaxFF MD) is used to study these interfacial phenomena at the (non-)reactive interface. The non-reactive interfaces are compared using a phenomenological theory (PT), predicting the temperature discontinuity at the interface. By connecting ReaxFF MD and PT we confirm a continuous temperature profile for the homogeneous non-reactive interface and a temperature jump in case of the heterogeneous non-reactive interface. ReaxFF MD is further used to understand the effect of chemical activity of two solids in contact. The selected Si/SiO_2_ materials showed that the TBR of the reacted interface is two times larger than the non-reactive, going from $1.65\times 10^{-9}$ to $3.38\times 10^{-9}$ m^2^K/W. This is linked to the formation of an intermediate amorphous layer induced by heating, which remains stable when the system is cooled again. This provides the possibility to design multi-layered structures with a desired TBR.

## 1. Introduction

**Figure 1:**
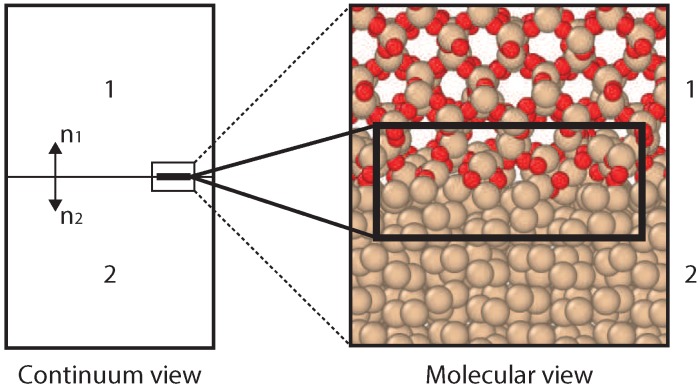
A schematic representation of continuum and molecular view of a system chosen for the present study.

Molecular characteristics of solids in contact play a key role in various fundamental studies related to heat transfer [1], mechanical behavior [2], micro/nano-fluidics [3], and catalysis [4]. Besides, it plays an important role in applications related to semiconductors [5], microelectronics [6,7] and heat-shielding in re-entry vehicles for aerospace applications [8]. In the latter case, insight into the thermal resistance at an interface of a multilayer structure is crucial for prediction and control of overheating of the thermal protection system used for the re-entry shuttle. The non-equilibrium effects of a hypersonic flow impinging on a solid interface requires detailed investigation of the boundary processes. Mass, momentum, energy transfer and chemical reactions on the interface are critical under these extreme conditions [9]. These processes can change the interfacial properties significantly compared to initial bulk properties. Furthermore, in case of chemical reactions at the interface (as shown in Figure 1), insight into the heat transfer in a small layer of material (a few molecular layers) is required.

The influence of the solid interfaces on thermal properties can be analyzed by the local temperature profiles on a molecular level. Experimental measurement of a temperature profile at the molecular level is extremely challenging. Therefore, computational models can be useful accurate tools to provide thermal insight and to establish the interfacial thermal correlations. In the context of building up a macroscopic theory of gas–surface interactions targeting the hypersonic re-entry flows, Giordano et al. [8] have proposed a Phenomenological-Theory (PT) to study heat transfer between two solids in contact. Another tool is Molecular Dynamics (MD) simulations, which has been used before to investigate thermal transport across solid interfaces [10,11,12,13,14,15,16], and significant influences of the solid interface on the thermal conduction are reported. A schematic representation of these two methods is shown in Figure 1, with a continuum view for PT, and a molecular view for ReaxFF.

Though temperature profiles at the solid interface are investigated before, these studies and methods focus mainly on non-reactive interfaces. However, chemical activity at the interfaces can influence the thermal behavior of solids in contact. Therefore, reactive force field Molecular Dynamics (ReaxFF [17,18]) is used, which is able to capture the chemical reaction and its influence on the surface transformation and temperature profile on molecular level.

In this study, we first consider the characterization of material properties like thermal expansion, thermal conductivity, and elastic properties using ReaxFF, to validate the force field. Thereafter, we build a generic non-reactive system, in which an interface is created based on the same material, for this, we considered two Platinum slabs (homogeneous Pt/Pt interface). Further, we create an interface between two different materials in contact, a non-reactive heterogeneous Platinum-Nickel interface (Pt/Ni). Accordingly, the temperature profiles from the ReaxFF MD simulations are compared with the macroscopic level PT based model of Giordano et al. [8]. For the computation of the temperature profile with PT, relevant material properties are computed with ReaxFF MD and upscaled, to be used as input in the PT model. Platinum and Nickel were selected because they are well studied, non-reactive, monatomic, and have similar lattice size.

After we analyzed this generic model, we created the reactive heterogeneous interface of Silicon and Silicon-oxide (Si/SO_2_). This Si/SiO_2_ interface is of relevance for many applications in the semiconductor industry, as well as aerospace engineering [19]. Because of its relevance, the Si/SiO_2_ interface is studied numerous times before [20], including the thermal boundary resistance (TBR) based on experiments [21], or numerical methods like MD [12,13,14], acoustic and diffusive mismatch models (AMM, DMM) [22], and phonon wave-package method [12]. With ranging values between 0.4–3.5 $\times 10^{-9}$ m^2^K/W, depending on the method and composition of the materials. Furthermore, Chen et al. showed a strong correlation between the coupling between the materials and the TBR. These coupling can be directly related to reactions happening at the interface. However, to our knowledge no systematic studies has been done on the influence of a reactive interface on the TBR. Therefore, we used ReaxFF to study the influence of the reactive interface. The Si/SiO_2_ system is kept at various temperatures, within the ReaxFF simulations, to increase/decrease the chemical activity. Accordingly, the TBR is computed. The TBR is defined as the temperature discontinuity at the interface (ΔT) divided by the heat flux (*Q*) that crosses the interface, see Equation (1). The TBR is often referred as the Kapitza resistance [23], however, we kept the analogy of Peterson et al. [24]:

(1) TBR=R=ΔT/Q

## 2. Methodology & Material Properties Estimation

### 2.1. Phenomenological-Theory (PT)

To study the heat transfer between two solids in contact, Giordano et al. [8] have proposed a Phenomenological-Theory (PT). With the aim of building up a macroscopic theory of gas–surface interactions targeting the hypersonic re-entry flows. They have remarked the lack of a physical principle justifying the standard temperature-continuity boundary conditions as a replacement of temperature-continuity, and have introduced tension continuity:

(2) $$n_{1}\cdot \tau_{U,1}\left(P,t\right)+n_{2}\cdot \tau_{U,2}\left(P,t\right)=0$$

where, $n_{1}$ and $n_{2}$ are normal unit vectors at a point of contact *P* and time *t*. This macroscopic theory is founded on momentum conservation and represents a more physically motivated boundary condition. For the mathematical formulation of the phenomenological-theory and further details, readers can refer to the original paper [8]. This method is used to compare the temperature profile non-reactive interfaces studied with MD. For the input of required material properties, the MD calculated values are used.

### 2.2. Reactive Force Field Molecular Dynamics

Molecular Dynamics (MD) is a computational method to obtain macroscopic and microscopic properties from approximated trajectories of individual particles. These approximated trajectories, obtained from Newton’s equations of motion, form an ensemble from which macroscopic properties of materials can be obtained [25]. To capture the chemical change during a reaction, Reactive force field (ReaxFF) [17] is used. ReaxFF is computationally more expensive than the non-reactive force field, however, it allows bond formation and bond breaking during the simulations, which makes simulations of chemical reactions possible. According to ReaxFF the bond order between a pair of atoms can be obtained directly from the inter-atomic distance, which relation is used to mimic chemical change. The feature of bond formation and breaking allows the user not to give predefined reactions pathways, these should present themselves given the right temperatures and chemical environment. However, the accuracy of this relies directly on the training set and the weights that are used to parameterize the reactive force field. Therefore, we tested several available ReaxFF on their ability to predict relevant material characteristics for our study. ReaxFF is widely used in studying chemical activities at a molecular level [18,26], including many Si/SiO_2_ systems [19,20,27,28,29,30,31,32,33,34,35].

For the in-silico characterization using ReaxFF MD methodology, we compute the material properties of relatively simple systems of Platinum (Pt), Nickel (Ni), Silicon (Si) and Silicon dioxide (SiO_2_). Furthermore, the elastic properties, thermal expansion coefficient, and thermal conductivity of the materials are important parameters for the phenomenological-theory, and thus the computed values are used as input parameters to upscale the molecular results up to the macroscopic level.

#### 2.2.1. Force Field Selection

Selection of an appropriate force field is very important for in-silico characterization. The calculations of elastic properties, thermal expansion coefficients and the radial distribution function (RDF), guide as a selection criterion for the appropriate force fields. Supercells of (5×5×5) Pt, (5×5×5) Ni, (3×3×3) Si and (4×4×4) SiO_2_ are created, containing approximately 400–800 atoms, with initial volumes of 7547, 5469, 7890, and 10,913 Å^3^, respectively. Periodic Boundary Conditions (PBC) are applied in all directions. The unit cells of Pt [36], Ni [37], Si [38] and SiO_2_ [39] are taken from experimental crystallographic information files.

**Figure 2:**
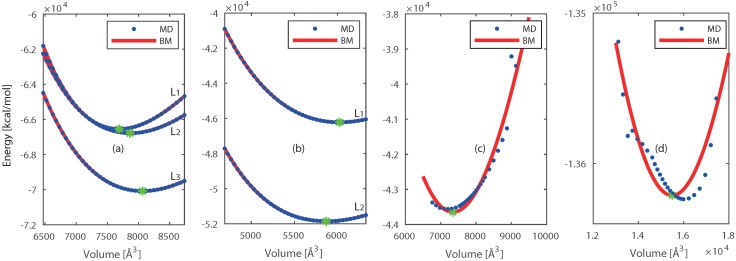
The energy of system obtained from selected force fields as a function of volume and the fitted BM-eos for: (**a**) 5×5×5 Pt, (**b**) 5×5×5 Ni, (**c**) 3×3×3 Si, and (**d**) 4×4×4 SiO_2_. The ‘☆’ represents the minimum energy point ($E_{0},V_{0}$) on each curve.

We have chosen three reactive force fields [4,29,40] available for Pt and Ni and nine force fields for Si and SiO_2_ [19,29,30,31,32,33,34,35]. These force fields are tested by deforming the crystals in the range of 0.86 to 1.16 times their initial volume. The resulting increase in potential energy is shown in Figure 2.

For clarity only the best performing force field is shown for Si and SiO_2_. The relation between volume and energy is found by integration of the pressure in the third order Birch-Murnaghan equation of state (BM-eos) [41,42,43], this relation is fitted to the results of the deformed crystal. The resulting parameters ($B_{0}$ and $V_{0}$) are given in Appendix A and compared with the literature.

The force field developed by J.E. Mueller et al. [4] showed the best results, and was therefore chosen for further use including Pt and Ni. This force field was parameterized for studying hydrocarbon reactions on nickel surfaces. They included the equation of state (EOS) for different Ni bulk structures in the training. Both our and their calculations predicted the EOS, together with the lattice parameters, in close agreement with quantum mechanical calculations. We predicted the equilibrium volume ($V_{0}$) of Pt [36] and Ni [37] unit cells within 1.9% and 7.0% deviations from their experimentally observed crystal structures. The deviations for the bulk modulus ($B_{0}$) of Pt and Ni are 9.8% [44] and 16.0% [45] respectively. Furthermore, Mueller et al. computed cohesive energies in close agreement with experimental values. The force field developed by Kulkarni et al. [19] was chosen for further use including Si and SiO_2_. This force field is an extension to include gas–surface reactions between oxygen and silica into an existed force field developed by van Duin et al. [20]. This original force field was parametrized to include the chemistry of silicon and silicon oxides, and the interface between these materials. Previous work of Tian et al. [46] also indicated that this original force field predicts the thermal conductivity of vitreous SiO_2_ in close agreement with experimental values. The force field of Kulkarni et al. is able to predict the equilibrium volume ($V_{0}$) within 7.5% [38] and 30% [39] deviations for Si and SiO_2_, respectively. The bulk modulus ($B_{0}$) is within 22.9% [47] and 5.7% [48] deviations for Si and SiO_2_, respectively. Therefore, this force field is selected for the study that includes Si and SiO_2_. These force fields are chosen for further investigation.

**Figure 3:**
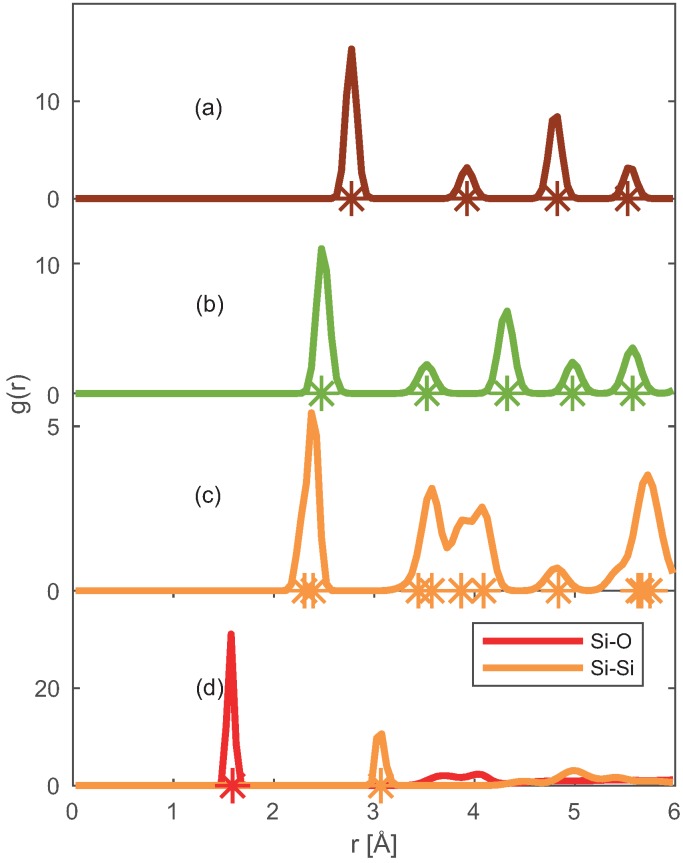
Radial distribution functions for: (**a**) Pt–Pt, (**b**) Ni–Ni, (**c**) Si–Si and (**d**) Si–O pairs present in studied solid crystals obtained from the ReaxFF MD simulations. The ‘*’ represents the neighboring atomic distances inside the solid crystal. For clarity, only the nearest neighbor ‘*’ is shown in SiO_2_.

To validate further the applicability of the chosen force fields, we have obtained the Radial Distribution Functions (RDFs) of Pt–Pt, Ni–Ni, Si–Si (Si and SiO_2_) and Si–O (SiO_2_) pairs from ReaxFF MD simulations in periodic solid supercells as shown in Figure 3.

The sharp peaks in RDF elucidate the extent of ordering in the supercell, thus representing the solid phase. The locations of the peaks coincide with the position of neighboring atoms (represented by ‘*’) in the experimentally observed solid crystal [36,37,38,39]. Concluding that the selected force fields [4,19] can capture the crystalline phase of Pt, Ni, Si, and SiO_2_.

The volumetric thermal expansion coefficient ($\alpha_{v}$) can be obtained from the slope of the natural logarithm of the volume (lnV) versus imposed temperature (*T*) [49]:

(3) $$\alpha_{v}=\frac{1}{V}{\left(\frac{\partial V}{\partial T}\right)}_{p}={\left[\frac{\partial ln(V)}{\partial T}\right]}_{p}$$

where $\alpha_{v}$ is the volumetric thermal expansion coefficient at constant pressure. We have varied the temperature over 250–500 K at atmospheric pressure in an NPT ensemble. The thermal expansion coefficients for Pt and Ni computed from ReaxFF MD and existing literature values are given in Table 1. The results on the molecular scale are in reasonable agreement with the bulk experimental value [50,51].

#### 2.2.2. Thermal Conductivity with Steady State NEMD Method

**Figure 4:**
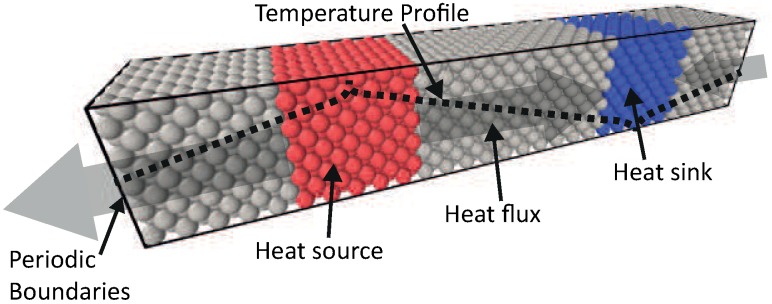
Schematic representation of the chosen system for steady state NEMD simulations. The heat source and sink are coupled to different temperatures with a damping constant of 100 fs. This will induce a heat flux through the intermediate zone, which is weakly coupled with a damping constant of 100.000 fs.

The thermal conductivity of a solid can be computed from Steady State Non-Equilibrium Molecular Dynamics (ss-NEMD). This method has been previously used [16,46,52,53,54,55], and it is based on imposing a temperature gradient over a system to estimate the thermal conductivity. A schematic view of this method is shown in Figure 4.

Two strongly coupled regions (using a Berendsen thermostat with damping constant τ = 100 fs) are created, one hot zone (red zone, $T_{H}$ = 330 K) and one cold zone (blue zone, $T_{C}$ = 300 K), which act as the heat source and sink, respectively. In between these two zones, there are weakly coupled regions (gray zone, τ = 10^5^ fs), this damping constant proved to have a negligible low influence on the system. This results in a steady state temperature gradient (dT/dx) and an energy flux (*q*), in the weakly coupled regions between heat source and sink.

From the energy flux and the temperature gradient, the thermal conductivity (*k*) can be computed, following Fourier’s law. This intuitive principle makes NEMD well suited to study thermal conductivity of different matter, and investigate the influence of structural defects and solid interfaces [52]. According to the kinetic theory, the thermal conductivity (*k*) is related to the mean free path (λ) of energy carriers (Equation (4)). If the characteristic length of the system is larger than the mean free path of carriers, thermal energy is transferred by multiple collisions. In this diffusive regime, the Fourier law is still valid. In cases when the characteristic length of the system is in the order of the mean free path, the energy carriers may travel ballistically between source and sink. This scattering in the heat source and sink introduces an extra limiting effect on the mean free path, and thus a reducing effect on the conductivity (Equation (4)). Thus, the conductivity equation must be corrected for the enhanced scattering effect [56]:

(4) $$k=\frac{1}{3}C_{v}v\lambda_{L}$$

where $C_{v}$ is the heat capacity, *v* the energy carrier velocity, and $\lambda_{L}$ the corrected mean free path for a system of size *L*. This can be estimated from Matthiessen’s rule [57], which states that the corrected resistivity is the sum of the intrinsic scattering and the scattering due to impurity. Thus, the corrected mean free path can be expressed as a combined effect of the mean free path of bulk ($\lambda_{\infty }$) and length of the system (*L*) as:

(5) $$\frac{1}{\lambda_{L}}=\frac{1}{\lambda_{\infty }}+\frac{4}{L}$$

In a system with periodic boundary conditions, the average distance for an energy carrier to scatter with the heat source or sink is L/4 [16]. Combining Equations (4) and (5), the thermal conductivity ($k_{L}$) of system size *L* can be expressed as:

(6) $$\frac{1}{k_{L}}=\frac{12}{C_{v}v}\frac{1}{L}+\frac{1}{k_{\infty }}$$

In this equation, $\frac{1}{k_{\infty }}$ is the thermal conductivity of the bulk material. Bulk thermal conductivity ($k_{\infty }$) can be estimated by extrapolating the effective thermal conductivity obtained for small system sizes ($k_{L}$).

The thermal conductivities of Pt and Ni are computed using the ss-NEMD method for different system sizes. The total length of the systems varied from 3×3×X (with *X* from 24 to 156), including 3×3×6 zones as the heat source and sink. The energy flux is taken from the average of the heat flux added and extracted by the two strongly coupled regions. The temperature gradient is computed over the weakly coupled region, and the system is equilibrated up to 1 ns with time steps (Δt) of 0.25 fs. The by ss-NEMD computed thermal conductivity values are presented in Table 2, and increases with system size for both Pt and Ni systems.

**Figure 5:**
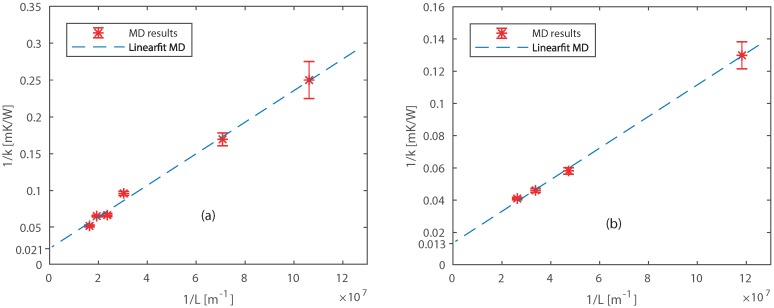
Extrapolation of thermal conductivities for different sizes of (**a**) Pt and (**b**) Ni.

The thermal conductivities are extrapolated for long length by fitting the linear expression between 1/L and 1/k (Equation (6)) as shown in Figure 5. The fitted lines intersect the Y axis at $1/k_{\mathrm{Pt}}$ = 0.021 mK/W and $1/k_{\mathrm{Ni}}$ = 0.013 mK/W, which results in a bulk thermal conductivity of $k_{\mathrm{Pt}}$ = 49.8 ± 10.5 W/mK and $k_{\mathrm{Ni}}$ = 74.4 ± 9.2 W/mK. The small deviations in an individual system result in large deviations in the bulk thermal conductivity, due to the extrapolation [59]. The computed thermal conductivities are approximately 35 % and 18 % lower than literature [58] values for Pt and Ni, respectively. This was expected because the ReaxFF formalism we used does not describe free electrons. The difference with experimental values can be explained by limitations of the ReaxFF method we used. ReaxFF MD is not able to model free electrons, thereby we underestimate the thermal conductivity. However, our aim is to compare models and confirm the temperature jump, not to compute exactly the thermal conductivities of Pt and Ni. There is a ReaxFF expansion including free electrons (e-ReaxFF) developed by Islam et al. [60]. At the moment of writing this e-ReaxFF concept does not include the studied materials, nonetheless, this might be interesting for future research.

The gradients of the extrapolated curve (Figure 5) obtained from ReaxFF MD simulations are 2.1 × 10^−9^ m^2^K/W and 1.0 × 10^−9^ m^2^K/W for Pt and Ni, respectively. The gradient should be equal to the theoretical gradient $12/C_{v}v$ (Equation (6)) obtained from the kinetic theory. By assuming a specific heat of $C_{v,\mathrm{Pt}}$ = 29 × 10^5^ J/m^3^K, and $C_{v,\mathrm{Ni}}$ = 40 × 10^5^ J/m^3^K [58], the computed velocities of thermal transport carriers are $v_{\mathrm{Pt}}\approx 2\;\times 10^{3}$ m/s, $v_{\mathrm{Ni}}\approx 3\;\times \;10^{3}$ m/s. These values are found to be in agreement with the speed of sound in the lateral direction through Pt and Ni from literature [58]. We also studied the final size effects perpendicular to the heat flow for platinum systems, see Appendix B. However, no finite size effects were observed for perpendicular directions, corresponding to the findings of Zhou et al. [54].

#### 2.2.3. Building the Interfacial Molecular System

**Figure 6:**
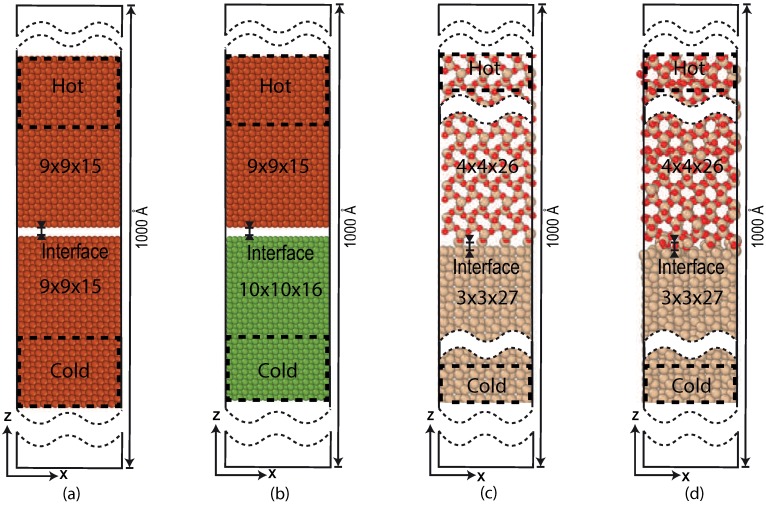
Schematic representation of systems: (**a**) non-reactive Pt/Pt interface, (**b**) non-reactive Pt/Ni interface, (**c**) initial reactive Si/SiO_2_ interface, and (**d**) the merged reactive Si/SiO_2_ interface. The particles in the dashed area are the strongly coupled sections, which acts as a heat source and heat sink.

Schematic diagrams of the studied Pt/Pt, Pt/Ni and Si/SiO_2_ systems are given in Figure 6. The crystal structures of Pt [36], Ni [37], Si, and SiO_2_ [39] are used to build the interfaces. For Si the bc8 form given by Kasper et al. [38] is used, and we used the cristobalite SiO_2_ of Nieuwenkamp et al., these two where specifically chosen to closely match each-others crystal lattice. The top and bottom sections (9×9×6 Pt, 10×10×6 Ni, 3×3×2 Si and 4×4×2 SiO_2_), are attached to strongly coupled thermostats (τ = 100 fs) and act as a heat source and heat sink. The intermediate regions (9×9×9 Pt, 10×10×10 Ni, 3×3×25 Si and 4×4×24 SiO_2_) are weakly coupled (τ = 10^5^ fs). The supercells are initially placed at a small distance and approach each other during an energy minimization to form the interface. From these energy minimized (merged) systems, the simulations are started. In the non-reactive systems, the top sections are kept at $T_{H}$ = 330 K and bottom sections are kept at $T_{C}$ = 300 K. In the reactive systems temperature values are varied to trigger a chemical reaction at the interface. To calculate the TBR, all the simulations are done over 1 ns, from which the last 0.75 ns are considered to obtain the data.

When an interface between two different materials is created artificial mechanical stresses are introduced by fitting the different lattice constants in one single periodic box. To restrict this to a minimum we carefully selected the materials, supercells, and orientation to create the interface. Thereby, the deformation of the crystals is limited to 0.1% compared to their literature value. Furthermore, we studied the influence of 1% deformation of Platinum on the thermal conductivity. The thermal conductivity for a compressed, as well as, a stretched crystal was lower, however for both cases within the standard deviation of the original system. The deformation of the crystals, and the study towards the thermal conductivity can be found in Appendix C.

## 3. Results

ReaxFF MD simulations are carried out to understand the temperature discontinuity across the solid interfaces of homogeneous (Pt/Pt), heterogeneous (Pt/Ni) and heterogeneous reactive materials (Si/SiO_2_). The computed material properties from the previous section are used to upscale the results from the molecular level to macroscopic phenomenological-theory level. For the thermal conductivity, the extrapolated value is used. A comparison is made between both methods for the non-reactive interfaces.

### 3.1. Non-Reactive Interfaces

**Figure A1:**
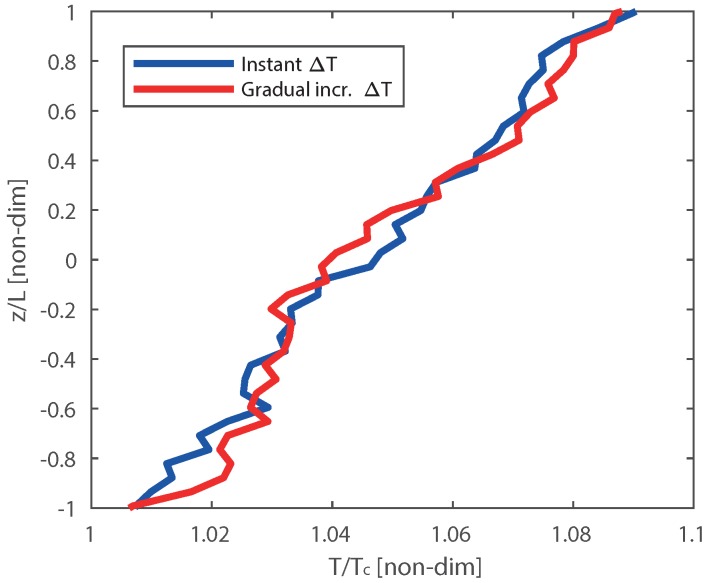
Comparison of temperature profiles (non-dim. *T* versus non-dim. *x*) obtained from ReaxFF-MD simulations for the Pt/Pt interface in the case where the heat source is immediately heated to $T_{H}$ = 330 K (instant Δ*T*) and system in which the heat source is gradually heated to $T_{H}$ = 330 K in steps of 5 K per 1 × 10^5^ iterations (gradual Δ*T*).

In realistic experimental conditions, the thermostat takes time to set the desired temperature, thus the temperature of the heat source evolves with time. To study this effect on the final temperature profile in ReaxFF MD, we compared two different settings to increase the temperature of the heat source. One with a gradual temperature increase to $T_{H}$, and one with an instantaneously high temperature at $T_{H}$. See Appendix D, and Figure A1, for the result of the comparison between the two cases. We observe that the final temperature profile is almost the same for both cases. Thus in the following cases, we have initialized the temperature of the heat source instantaneously at high temperature (instant Δ*T*).

**Figure 7:**
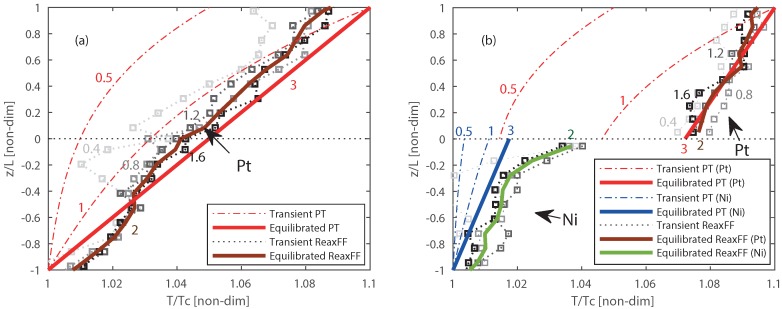
Comparison of temperature profile computed from PT based model and ReaxFF MD simulation for: (**a**) Pt/Pt interface. (**b**) Pt/Ni interfaces. The solid lines represent the equilibrated, and the dashed/dotted lines the transient temperature profiles. The dashed-dotted lines are intermediate transient temperature profiles for PT, and the dotted lines with square markers represent intermediates from ReaxFF MD simulations. The corresponding numbers at the lines represent $t_{\text{non-dim}}$.

For the non-reactive ReaxFF MD interface investigation, the systems given in Figure 6a,b are studied. The temperature profile evolution across the solid interfaces of Pt/Pt and Pt/Ni systems with ReaxFF MD is plotted in gray-scale after every 200 ps, which can be seen in Figure 7a,b. The light-gray to black lines represents respectively the earlier and later time periods.

The equilibrated temperature is represented by the solid brown and green lines, respectively Platinum and Nickel. The temperature profiles developed over time obtained from ReaxFF MD are compared with the temperature profile from phenomenological-theory as shown in Figure 7. The molecular level ReaxFF MD simulations and macroscopic level phenomenological-theory have a different time scale, thus to compare them, a non-dimensional time ($t_{\text{non-dim}}$) is defined as:

(7) $$t_{\text{non-dim}}=t/t_{eq}$$

where *t* is the actual time and $t_{eq}$ is the time assumed the system is in steady state. An steady state time of 0.5 ns is assumed for the MD simulations. The red, blue and gray numbers in the figures represent these different transient $t_{\text{non-dim}}$ states. The temperature profile obtained from the phenomenological-theory evolves slowly with time when compared with the ReaxFF MD simulations, where the transient states are quickly converging and fluctuation around the equilibrium state. In the ReaxFF MD results, a continuous temperature profile is observed at the Pt/Pt interface while a temperature jump (discontinuity) is observed at the Pt/Ni interface as shown in Figure 7a,b, respectively. Similar temperature profiles are also observed from the phenomenological-theory. The ReaxFF MD method shows a temperature jump of approximately 39%, where the phenomenological-theory results in a 55% jump of the imposed temperature difference of 30 K. Since one method is based on molecular level and another one is based on macroscopic level theory, a slight discrepancy in the magnitude of the temperature jump can be expected. These results confirm that the temperature jump is observed at the solid interface between different materials for both molecular level and macroscopic level modeling.

### 3.2. Reactive Interfaces

**Figure A2:**
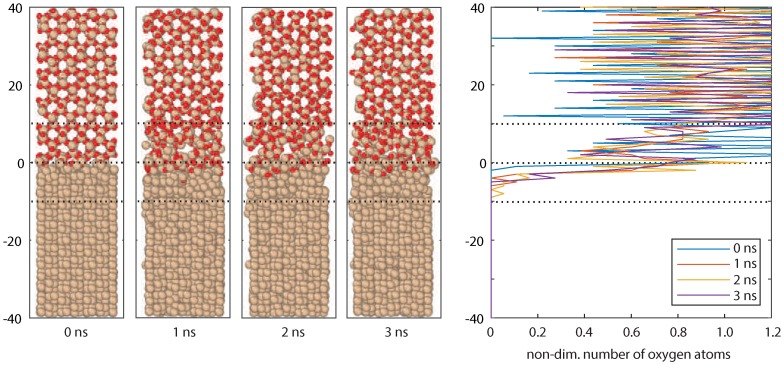
Snapshots of the Si/SiO_2_ interface.

At the surface of re-entry vehicles chemical reactions can occur, these reactions contribute to the heating of such vehicles [8,19]. Furthermore, these reactions form a small layer, and influence the heat and mass transport at the surface. To gain more fundamental knowledge of such a surface, we studied a reactive solid Si/SiO_2_ interface (see Figure 6c,d). The building of the physical system is similar to the two previous systems (Pt/Pt and Pt/Ni). The length between the heat source and sink is approximately 327 Å, and temperatures of the heat source and sink are varied to increase/decrease the chemical reaction at the solid interface [61]. Four different cases are studied, for the first case (Case 1) the set temperatures for heat source ($T_{H}$) and sink ($T_{C}$) are 150 and 100 K, respectively. For the second case (Case 2), the temperatures are $T_{H}=$ 350 K and $T_{C}$ = 300 K, and for the third case (Case 3) the temperatures are $T_{H}$ = 850 K and $T_{C}$ = 800 K. These cases have an interface temperatures of approximately 125, 325, and 825 K. In the fourth case (Case 4) the complete system was heated to a high temperature (1700 K) for 3.5 ns to create a reactive region, and thereafter, cooled back to $T_{C}$ = 100 K, and $T_{H}$ = 150 K to stop the chemical activity completely again. After the cooling, a new steady state simulation was done at $T_{C}$ = 100 K, and $T_{H}$ = 150 K. From Figure A2 in Appendix E, one can see that the major part of the interface formation takes place within the first nanosecond. In terms the thickness of the interface, as well as, the depth of the oxygen penetration into the silicon surface only little changes are observed after the first nanosecond. Therefore, it was not needed to go for longer simulations to create the interface. This fourth case was chosen to get a distinct comparison between the non-reacted and reacted interface, at the same temperature (Case 1 and 4).

**Figure 8:**
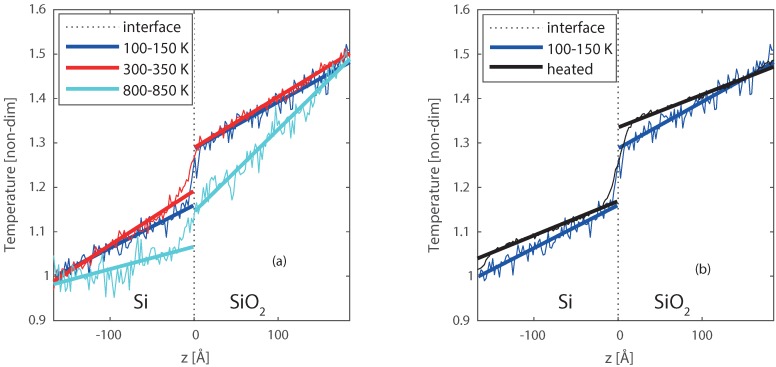
Temperature profiles for the Si/SiO_2_ interfaces, with (**a**) case 1, case 2, and case 3, and (**b**) case 1, and case 4. The dotted line indicates the location of the interface, the dark blue lines represent case 1 ($T_{H}$ = 150 and $T_{C}$ = 100 K), the red lines represent case 2 ($T_{H}$ = 350 and $T_{C}$ = 300 K), the light blue lines represent case 3($T_{H}$ = 850 and $T_{C}$ = 800 K), and the black line represents case 4 (first heated to 1700 K, and then a steady state at $T_{H}$ = 150 and $T_{C}$ = 100 K).

The resulted temperature profiles for Case 1–3 are plotted in Figure 8a. The temperature is made non-dimensional by taking 100–150 K as reference and divide by 100. The thick solid lines are fitted to the data, and extrapolated to the interface, to get the temperature jump. The initial interface is positioned at *z* = 0 Å in the figures. A temperature jump is observed between Si and SiO_2_ for all the cases. It reiterates that there is a temperature discontinuity at the reactive heterogeneous solid interface as well. Case 1 (100–150 K) shows a clear jump, with a sharp temperature profile. When the temperature is increased to 300–350 K (case 2), the jump remains, however, it is less sharp. This is caused by some small deformation at the interface induced by the temperature. Moving to even higher temperatures 800–850 K (case 3), not only a temperature jump but also a drop of the temperature profile over the entire system is observed. This suggests a heat sink at the interface, due to energy consumption by the deformation of the crystals at the interface. This deformation has an impact on the heat transfer and results in an intermediate region between the Silicon and Silica crystals of a few ångströms.

**Figure 9:**
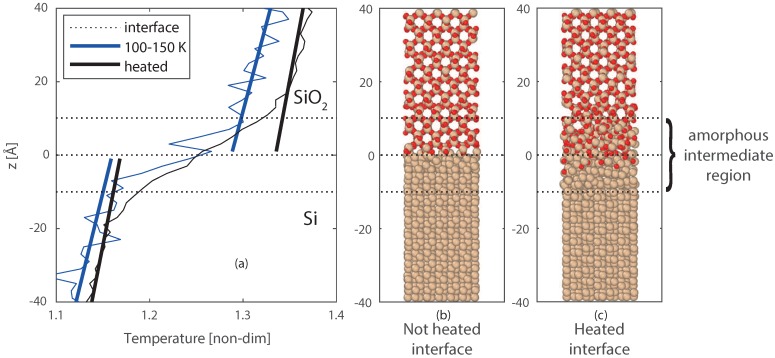
(**a**) zoom of Figure 8b, with the temperature profiles for case 1 ($T_{H}$ = 150 and $T_{C}$ = 100 K), and case 4 (first heated to 1700 K, and then a steady state at $T_{H}$ = 150 and $T_{C}$ = 100 K). The final molecular structures of the interfaces are given in (**b**) for case 1, and (**c**) for case 4.

To better study the effect and size of the intermediate region, the entire Si/SiO_2_ system was heated up to 1700 K to increase the reactivity and advance the formation of the intermediate region. Higher temperatures were also tested, however, these resulted in melting of the Silicon crystal, and separation of the two slabs. Lower temperatures would require more time to create a similar intermediate region. The heating process results in a larger intermediate amorphous region, where oxygen diffused up to 5 Å into the Silicon crystal, and deformation of both materials is visible up to 10 Å from the interface. After the heating, the system was cooled back to 100–150 K, this temperature was chosen to stop the chemical activity as far as possible. The final equilibrated temperature profile is compared with the temperature profile of case 1, where no reactive region is had been present at the interface. This comparison shown in Figure 8b, and a closer profile around the interface is shown in Figure 9a–c. From Figure 9c, the thicker interfacial region for the reacted interface can be clearly observed, compared to the non-reacted clean interface (Figure 9b). For the heated interface (case 4), the temperature jump (ΔT) is larger, however, is has become less sharp than case 1 and smoothed over the formed intermediate region.

The thermal boundary resistances (TBR) are given in Table 3. The calculated value for the low temperature (case 1) is in good agreement with Deng et al. [12], who found a value of $1.48\;\left(\pm 0.46\right)\times \;10^{-9}$ m^2^K/W using NEMD, and $1.37\;\left(\pm 0.42\right)\times 10^{-9}$ m^2^K/W using phonon wave-package dynamics approach. The higher temperatures (case 2,3) approach the experimental results of Hurley et al. [21], who measured a resistance of $2.3\times 10^{-9}$ m^2^K/W. The reacted interface, which includes an amorphous SiO_2_ region, is in good agreement with the DMM results of Hu et al.[22], who found a resistance of $3.5\times 10^{-9}$ m^2^K/W, for amorphous SiO_2_ with Si. The temperature jump at the interface for the 300–350 K and 800–850 K temperatures (case 2,3) is smaller, however, the calculated TBR is higher, caused by the deformation of the interface which acts as an extra heat sink. The TBR for the reacted interface (case 4) is more than twice the TBR of the non-reacted clean interface (case 1) at the same temperature, caused by the amorphous Si–SiO_2_ intermediate region.

## 4. Conclusions

ReaxFF MD is known to capture the physical and chemical phenomena under various conditions [18,26]. We have chosen various force fields for Pt/Ni and Si/SiO_2_ systems, which can mimic their material properties. The selected force fields predict the equilibrium volumes [36,37,38,39] and bulk modulus [44,45,47,48] of respectively Pt, Ni, Si and SiO_2_ in close agreement with experiments/theory. To validate further, thermal expansion and thermal conductivity coefficients of Pt and Ni are estimated using ReaxFF MD. The thermal expansion coefficients are found to be in reasonable agreement with experiments. The thermal conductivity of a solid material is size-dependent on the molecular level. Thus, we have obtained the thermal conductivity of Pt and Ni for various system sizes and extrapolated to a very long length to determine the bulk thermal conductivity.

The elastic and thermal properties, obtained from ReaxFF MD, served as input parameters for the macroscopic level phenomenological-theory (PT) [8]. Temperature profiles of non-reactive interfaces, obtained from both methods, are compared. In this comparison, we have reported the temperature profiles across a homogeneous (Pt/Pt), and heterogeneous (Pt/Ni) interface. Temperature continuity is observed at the solid homogeneous interface of Pt/Pt. The temperature profile of the molecular level simulation is faster at equilibrium than the phenomenological-theory. The temperature profiles between Pt/Ni has a discontinuity at the interface observed in both molecular and macroscopic level. The temperature jump obtained from the molecular level calculation is 18% lower than the one obtained from PT calculations. The discrepancy between the two models in the temperature jump for Pt/Ni is minimal, and can be explained by the fact that the length- and time-scale for both calculations are different, and/or the length dependence of the thermal conductivity. We can conclude that both models, the molecular level ReaxFF MD simulations and the PT, predict a temperature discontinuity across the solid boundary if the materials are not the same.

The ReaxFF MD methodology can capture chemical reactions, therefore, interesting insights could be obtained for solid pairs which can form a reactive interface. For this purpose, the Si/SiO_2_ pair was chosen and the heat source and heat sink were varied to increase/decrease the chemical reaction at the interface. Three distinct solids (Si, amorphous reacted Si–SiO_2_ interface, and SiO_2_) have been observed. The thermal boundary resistance (TBR) is computed at the Si/SiO_2_ interface for the different systems, providing us with information of the TBR over interfaces with different chemical activity. It can be concluded that the reacted amorphous region at the interface introduces extra resistivity, compared to the non-reactive clean interface. Showing the opportunity to control the thermal resistivity of a multi-layered system by controlling the interfacial reactive regions.

## Figures and Tables

**Table 1:** Comparison of volumetric expansion coefficients ($\alpha_{v}$) obtained from the present ReaxFF MD simulations, and reported from literature (in parentheses).

Element	$\alpha_{v}\times 10^{-5}$ (m^3^/m^3^K)
Pt	2.0 (2.7 [50])
Ni	3.2 (3.9 [51])

**Table 2:** Thermal conductivities for different system sizes of Pt and Ni.

System	Conductivity Pt (W/mK)	Conductivity Ni (W/mK)
3 × 3 × 24	4.0±0.4	7.7±0.5
3 × 3 × 36	5.9±0.3	—
3 × 3 × 60	—	17.2±0.6
3 × 3 × 84	10.4±0.3	21.7±0.6
3 × 3 × 108	15.0±0.5	24.4±0.5
3 × 3 × 132	16.3±0.3	—
3 × 3 × 156	19.2±0.5	—
Extrapolated	49.8±10.5	74.4±9.2
Literature	71.6 [58]	90.7 [58]

**Table 3:** Thermal Boundary Resistance (TBR) of Si/SiO_2_ systems, with $T_{C}$ and $T_{H}$ (K) as thermostatted temperature for the heat sink and source respectively.

Case	$T_{C}$ (K)	$T_{H}$ (K)	Temperature Jump, ΔT (*K*)	TBR $\left(\frac{m^{2}K}{W}\right)$
1	100	150	12.8	$1.65\times 10^{-9}$
2	300	350	9.7	$2.40\times 10^{-9}$
3	800	850	7.8	$2.34\times 10^{-9}$
4	1700 → 100	1700 → 150	16.6	$3.38\times 10^{-9}$

## Appendix A. Birch-Murnaghan Equation of State Fitting Si & SiO_2_

To select the correct force field, a BM-EOS study is done. The results of the fitting are presented in Table A1, with $r^{2}$ as correlation coefficient of the fitting. The results are compared with literature, which gives $V_{0,\mathrm{Pt}}=60.38\;{Å}^{3}$ [36], $V_{0,\mathrm{Ni}}=43.76\;{Å}^{3}$ [37], $V_{0,\mathrm{Si}}=292\;{Å}^{3}$ [38], $V_{0,{\mathrm{SiO}}_{2}}=171\;{Å}^{3}$ [39], $B_{0,\mathrm{Pt}}=266$ GPa [44], $B_{0,\mathrm{Ni}}=185$ GPa [45], $B_{0,\mathrm{Si}}=98$ GPa [62], and $B_{0,{\mathrm{SiO}}_{2}}=36$ GPa [48]. The ReaxFF of Mueller et al. [4] and Kulkarni et al. [19] proves to be the best applicable for respectively the Pt/Ni system and the Si/SiO_2_ system.

**Table A1:** Results of BM-eos fitting to different ReaxFF.

	Reference Force Field	$B_{0}$ (GPa)	$V_{0}$ (Å^3^)	$E_{0}$ (kcal/mol)	$r^{2}$	Figure
Pt	[4]	240	61.52	−532.4	1.0	Figure 2a L1
	[40]	179	62.91	−534.3	1.0	Figure 2a L2
	[29]	166	64.52	−560.6	1.0	Figure 2a L3
Ni	[4]	155	46.96	−414.9	1.0	Figure 2b L2
	[29]	167	48.21	−369.8	1.0	Figure 2b L1
Si	[19]	144	272.1	−1617	0.92	Figure 2c
	[30]	165	273.6	−1611	0.88	
	[31]	292	252.9	−1729	0.95	
	[35]	216	291.2	−1675	0.53	
	[29]	295	265.7	−2244	0.84	
	[63]	235	268.3	−2206	0.95	
	[34]	334	255.2	−1728	0.95	
	[33]	289	252.3	−1730	0.95	
	[32]	334	255	−1728	0.95	
SiO_2_	[19]	35	242.1	−2128	0.83	Figure 2d
	[30]	500	244.6	−1847	0.60	
	[31]	33	260.0	−1818.6	0.86	
	[35]	34	238.1	−1793	0.98	
	[29]	47	244.7	−1860	0.83	
	[63]	234	247.2	−1828	0.78	
	[34]	331	263.4	−1841	0.19	
	[33]	273	258.1	−1837	0.16	
	[32]	331	263.4	−1840	0.19	

## Appendix B. Finite Size Effects, Perpendicular to the Heat Flow

Platinum systems (3×3×32, 5×5×32, 10×10×32) were used to study the finite size effects in perpendicular direction to the heat flow. The system are placed in vacuum in z-direction and have periodic boundary conditions in x- and y-direction, the given crystal sizes are including heat source and sink. No final size effects are observed in perpendicular direction to the heat flow.

**Table A2:** Thermal conductivity of Pt-systems in vacuum in z-direction, and different sizes in x- and y-direction.

System	Thermal Conductivity (W/mK)
3×3×32	9.7
5×5×32	8.0
10×10×32	10.7

## Appendix C. Influence of Mechanical Deformation of Slabs

When a heterogeneous interface with two different materials, and thus different lattice parameters, is created the materials are compressed or stretched to fit both materials within the same periodic box. This introduces extra mechanical stresses in the crystals. To restrict this to a minimum we have chosen the materials and supercells in such a way that these artificial deformations are kept to a minimum. The lattice parameters are given in Table A3 and Table A4 for the literature value, after an energy minimization in ReaxFF, after an energy minimization in ReaxFF with a vacuum in z direction, and the size used in this work. The lattice parameters are compared with the literature value and the error is given in the last column, this shows that the values are all within 3%, and there is only 0.1% deformation in this work compared to the lattice from literature.

**Table A3:** Lattice constant for Pt and Ni.

System	Lattice [a; b; c] (Å)	Deviation from Literature (%)
Pt-literature [36]	3.9231; 3.9231; 3.9231	-
Pt-EM ReaxFF	3.9473; 3.9473; 3.9473	+0.6
Pt-EM ReaxFF + vacuum in z-direction	3.9412; 3.9412; —	+0.5
Pt-interface	3.9192; 3.9192; —	−0.1
Ni-literature [37]	3.5238; 3.5238; 3.5238	—
Ni-EM ReaxFF	3.6122; 3.6122; 3.6122	+2.5
Ni-EM ReaxFF + vacuum in z-direction	3.6048; 3.6048; —	+2.3
Ni-interface	3.5273; 3.5273; —	+0.1

**Table A4:** Lattice constant for Si (bc8), and cristobalite SiO_2_.

System	Lattice [a; b; c] (Å)	Deviation From Literature (%)
Si (bc8)-literature [38]	6.636; 6.636; 6.636	—
Si (bc8)-EM ReaxFF	6.4393; 6.4393; 6.4393	−2.9
Si (bc8)-EM ReaxFF + vacuum in z-direction	6.4383; 6.4046; —	−2.9; −43.5
Si (bc8)-interface	6.6273; 6.6273; —	−0.1
SiO_2_-literature [39]	4.964; 4.964; 6.920	—
SiO_2_-EM ReaxFF	5.0443; 5.0443; 7.0063	+1.0
SiO_2_-EM ReaxFF + vacuum in z-direction	5.0443; 5.0443; —	+1.0
SiO_2_-interface	4.9705; 4.9705; —	+0.1

### Appendix Influence of Stress on Thermal Conductivity

To form an interface with different materials, the materials are slightly stressed to match each other lattice constants. One of the two materials was slightly compressed, and the other one slightly stretched to form the interface. In the Table A3 and Table A4 amount of deformation is shown for the materials used in this work, which are within ±0.1%. To gain more knowledge on the effect of these stresses on the heat transport across the material, we computed the thermal conductivity for 3 × 3 × 132 Platinum structures with lattices corresponding to the literature value, 1% compressed structures, a 1% stretched structures. The computed thermal conductivities for the different deformation are given in Table A5, and are within each other’s standard deviation. Thereby, we conclude that we can neglect the effect of stress in this study.

**Table A5:** Thermal conductivity of a 3 × 3 × 132 Platinum structure under different mechanically induced stresses.

Simulation	No Stress k (W/mK)	1% Compression k (W/mK)	1% Stretched k (W/mK)
1	15.2	14.6	13.4
2	17.8	13.9	14.6
3	16.7	15.3	17.4
4	15.6	16.5	16.5
Average	16.3 ± 1.2	15.1 ± 1.1	15.5 ± 1.8

## Appendix D. Comparison of Gradual and Instant Induced Temperature

In realistic experimental conditions, the thermostat takes time to set the desired temperature, thus the temperature of the heat source evolves with time. To investigate the effect of heating the systems on the final temperature distribution in ReaxFF-MD, we have compared a gradual temperature increase to $T_{H}$ and an instantaneously temperature at $T_{H}$. For the gradual temperature setting, we have increased the temperature of the heat source ($T_{H}$ = 330 K) in the steps of 5 K per 0.1 million iterations (25 ps). After 0.6 million iterations (150 ps), the temperature profile of the gradual temperature rise system is compared with the system in which temperature of hot zone was instantaneously set at $T_{H}$ = 330 K (instant Δ*T*) as shown in Figure A1, the comparison was done over a total range of 1 million iterations. We observe that the equilibrated temperature profile is almost the same for both cases. Thus in the following cases, we have initialized the temperature of the heat source instantaneously at high temperature (instant Δ*T*).

## Appendix E. Development of the Amorphous Si/SiO_2_ Interface

To create a reactive formed interface between Si and SiO_2_, the system was heated for to high temperatures (1700 K) for 3.5 ns. Thereafter, it was cooled back to $T_{C}$ = 100 K, and $T_{H}$ = 150 K to stop the chemical activity completely again. The development of the interface over the first 3 ns can be observed in the snapshots in Figure A2, also the number of oxygen atoms are counted for these snapshots.
